# Mothers of adolescent girls and Human Papilloma Virus (HPV) vaccination in Western Kenya

**DOI:** 10.11604/pamj.2021.38.126.21359

**Published:** 2021-02-04

**Authors:** Hillary Mabeya, Jack Odunga, Davy Vanden Broeck

**Affiliations:** 1 Moi University, Eldoret, Kenya,; 2 International Center of Reproductive Health, Ghent University, Ghent, Belgium,; 3 Ghent University, Ghent, Belgium

**Keywords:** Human papilloma virus, vaccination, adolescent girls, Western Kenya

## Abstract

**Introduction:**

human papilloma virus (HPV) which is preventable is the main cause of cervical cancer and it targets mostly young adolescents. The study was to determine the practice desire, attitude and knowledge of mothers of adolescent girls on HPV vaccination in Western Kenya.

**Methods:**

this was a descriptive cross-sectional study design. Data was obtained using semi-structured questionnaires and analyzed using both descriptive and inferential statistics at 95% confidence level using the SPSS software version 22. A p-value ≤ 0.05 was considered statistically significant.

**Results:**

ninety five percent of the mothers had intentions to vaccinate their daughters and also had a positive attitude and their response to HPV vaccination was significantly lower than those without intentions p=0.02, 95% CI, OR=0.48 (0.90-0.89). Vaccination against HPV was low at 9.4% with a mean age of 34 years. Our results found a high level of cervical cancer awareness (85.0%), HPV and vaccine awareness respectively (62.0%, and 64.0%). “Vaccination of my daughters will prompt early sexual activity and the cost of HPV vaccination being a barrier to vaccination” had a statistically significant influence on the practice of vaccination. Negative attitude to daughters´ early onset of sexual activity significantly reduced up take while positive attitude to cost of HPV vaccine significantly increased up take of HPV vaccination with p value of 0.007 and 0.04 respectively.

**Conclusion:**

awareness of HPV and HPV vaccine prevention is low among mothers of adolescent girls in Western Kenya. There was a positive attitude and high desire towards the use of HPV vaccination therefore a need for awareness, policy and unify efforts to reduce cervical cancer burden.

## Introduction

Cervical cancer related deaths in 2018 accounted for 266,000 (7.5%) of female cancer death worldwide with an estimated 528,000 new cases annually. In Kenya, cervical cancer is the second most frequent cancer after breast cancer and accounts for an average of 2000 deaths per year. Kenya has a crude incidence rate of 16.5 per 100,000 women and age standardized rate of 28.7 [1, 2]. The World Health Organization (WHO) promotes concerted approach to cervical cancer prevention and control to identify opportunities to deliver effective involvement of all stakeholders [3]. Cervical cancer prevention involves a combination of knowledge and other advancements in several areas in an effort to promote cervical cancer prevention and treatment [4, 5].

Human papilloma virus (HPV) is the most widespread sexually transmitted infection and studies have shown that 50-80% of sexually exposed are infected at least once in their life time [6, 7]. HPV 16 and 18 are commonest oncogenic type and have been implicated in 60-78% of squamous cancer of the cervix and 72-94% of cervical adenocarcinoma [8]. Prevention of cervical cancer is emphasized on early recognition and diagnosis of precancerous lesion of the cervix until the development and introduction of HPV vaccines that offers prospect for primary prevention [9, 10]. These vaccines have approximately total protection against new and constant infection and are recommended for girls between the ages of 9 years to 14 years [11-13].

Villa *et al*. (2005) and Harper *et al*. (2006) in their researches on prophylactic quadrivalent human papillomavirus (types 6, 11, 16, and 18) posited that the most effective preventive method against cervical cancer among adolescent girls and other women prior to sexual exposure is the primary prevention by the HPV vaccination [14, 15]. Therefore, World Health Organization recommends giving HPV vaccine to girls between the ages of 9-14 years, prior to sexual exposure, since the vaccine will be more efficient if girls have not already acquired the HPV virus [16]. There is need for Kenya's Ministry of Health to target girls between the ages of 9-15 years for HPV vaccination [17]. HPV vaccination in Kenya is currently being provided by the government in a free mass immunization program launched in the year 2019. There is paucity of data on information about HPV vaccination. Health management information is a major limitation as majority of the adolescent girls and their mothers have limited access to health information.

HPV vaccination has been recommended for introduction into national immunization programs in various countries. HPV vaccine has been incorporated into the school vaccination program for young adolescent girls in some developed countries which include: USA, Australia, New Zealand and Sweden. This practice however faces the challenges which are common to new vaccines. Parental consent is needed for the vaccination of minors, for this reason, parental knowledge, attitude to the vaccine and acceptance to vaccinate their daughters would to a good extent determine the success of the HPV vaccination program. Factors influencing practice of HPV vaccination such as: vaccine awareness, child age, perceived access to the vaccine, societal norms, religious inclination, stigmatization against sexually transmitted infection, vaccine safety and suspect of potential long-term adverse outcome, perception about disease susceptibility and severity, interaction with clinicians, the need to involve the adolescent in decision and mother´s cervical dysplasia and cervical cancer experience are thought to influence the uptake of HPV vaccination in Kenya.

The progress in the HPV vaccination programs and awareness creation about vaccination has remained slow in Kenya. Few studies in our environment have investigated the disposition, attitude, intention to vaccinate and practice of HPV vaccination of adolescent girls by their mothers. Few studies have evaluated the acceptability of the HPV vaccine in Kenya and we found no such studies in Eldoret in particular. Hence this study aimed at determining the knowledge, attitude, desire and practice of HPV vaccination of adolescent girls and factor that determine the intent and practice of HPV vaccination of adolescent girls by their mothers in Eldoret. In order to achieve the purpose of this study, the following research questions were answered: what is the extent of the practice of adolescent girls HPV vaccination by mothers in Eldoret? what is the level of Knowledge on cervical cancer, HPV and HPV vaccine? what is the attitude of mothers towards vaccination of their adolescent daughters? and what are the factors influencing practice of HPV vaccination among adolescent girls?

## Methods

This was a questionnaire-based cross-sectional survey carried out in Eldoret, Uasin Gishu County in Kenya at Moi Teaching and Referral and Gynocare Womens and Fistula Hospital. The study was conducted between January 2018 and July 2018. Eldoret is a principal city in Western Kenya. It is a capital of Uasin Gishu County with the location elevation varies from about 2100 metres above the sea level to more than 2700 metres (7000-9000 feet). Gynocare Womens and Fistula Hospital is a 100 bed hospital dedicated to fistula surgery, gynecological and obstetric care services. The hospital offers cervical cancer care. Moi Teaching Hospital is a 1000 bed national hospital dedicated to teaching and referral hospital in Eldoret. The reproductive health department has 45 beds dedicated to gynecology and 150 beds dedicated to obstetrics.

The study was based on convenience sampling of consenting mothers who accompanied their daughters to gynecological and adolescents clinics. The study population included randomly selected mothers of adolescent girls as at the time of the administration of questionnaire aged above 18 years. Random sampling was used to determine the sample size and recruitment of the study participants. Random sample generator was used until the desired sample size was achieved.

Face-to-face structured interviews were conducted by trained research assistants after the purpose of the study had been explained to the participants and their informed consent sought and gained. Women who did not give their consent were excluded from this study. Ethical approval was sought and given by the Institutional Research and Ethics Committee (IREC) and the Moi Teaching and Referral Hospital. IREC/2017/120.

A standardized questionnaire assessing knowledge of HPV, cervical cancer, pap smear test, vaccine acceptability, and willingness to participate in HPV vaccination as well as demographic characteristics related to HPV and cervical cancer was administered to a total of 300 mothers of adolescent girls. The questionnaire was explained to those participants who could not read and understand English. This questionnaire consisted of 5 sections seeking information about the socio-demographic characteristics of the respondents such as age, marital status, religion, occupation and educational attainment, their knowledge of cervical cancer, HPV infection and HPV vaccination, and their attitude and practice of HPV vaccination of the daughters.

The study was tested for validity: construct validity; establishing correct operational measures for the concepts being studied. Internal validity; for explanatory and casual studies only, not for descriptive or explanatory studies establishing a casual relationship, thereby certain conditions are shown to lead to another conditions. The depth of knowledge in the questionnaire was determined by grading respondent´s responses on knowledge into poor, good and excellent. Five points Likert´s scale was used to assess attitude; where various degrees of attitude were assessed using options of 1) strongly disagree; 2) disagree; 3) undecided; 4) agree; 5) strongly agree.

In addition, the questionnaire was tested for reliability by using cronbach coefficient alpha to determine the internal consistency of the items. This method was used for estimating reliability of test scores by the use of a single administration of a test. Therefore, provided good measures of reliability because holding other factors constant, the more similar the test content and conditions of administration are, the greater the internal consistency reliability (Mugenda and Mugenda, 2003). Data was computed and analyzed using statistical software SPSS for windows version 22. Data was analyzed using descriptive and inferential statistics. Frequencies and percentages were determined where appropriate. Statistical significance was determined using p-value. P-value of 0.05 was considered statistically significant.

## Results

**Demographic characteristics of respondents:** we sought to ascertain the demographic characteristics of the respondents and compare it with their knowledge, attitude, desire and practice of HPV vaccination on mothers and their adolescent girls and factors that determine the intent and practice of HPV vaccination of adolescent girls in Eldoret (Table 1). The respondent mothers were administered with questionnaire and interviewed. Table 1 shows some demographic data of respondents. A total of three hundred questionnaires (300) respondents who completed their questionnaires out of 310 who were administered with questionnaires giving a response rate of 96.7%. The mean age of the respondents was 34 years. Majority of the respondents were married, (88%) were Christians while the rest (12.0%) were Muslims; (73%) of the respondents were formally employed while (27.0%) were unemployed; and (15.0%) had primary education, (85.0%) had secondary/tertiary (post primary) education while (20.0%) had no education at all. This study showed that, socio-demographic characteristics of individuals such as age (p=0.039), marital status (p=0.0), occupation (p=0.0), level of education (p=0.002), and religion (p=0.00) have significant effects on their willingness to participate in research. Table 2 shows the awareness and level of knowledge of cervical cancer, human papilloma virus and HPV vaccine. Most of the respondents 85.0% were aware of cervical cancer, while 60.0% and 62.0% were aware of human papilloma virus and human papilloma vaccine respectively. Majority of the respondents were willing to definitely accept the HPV vaccine 70.0% while 30.0% were not willing to accept the HPV vaccine.

**Table 1:** socio-demographic characteristics of the respondents

Age	Frequency N=300	Percent
<30	38.0	27.2
30-39	32.9	44.7
40-49	21.4	21.4
≥50	7.8	5.7
**Marital Status**		
Married	234	78.0
Single	69	22.0
**Religion**		
Christian	264	88.0
Muslim	36	12.0
Others		-
**Occupation**		
Employed	219	73.0
Unemployed	81	27.0
**Level of Education**		
None	30	10.0
Primary	15	5.0
Secondary/Tertiary (Post primary)	255	85.0

**Table 2:** knowledge of cervical cancer and HPV and HPV vaccine

No	Items	Frequency N=300	Percent
**Awareness of cervical cancer**			
1	Yes	255	85.0
2	No	45	15.0
**Awareness of HPV**			
1	Yes	180	60.0
2	No	120	40.0
**Awareness of HPV Vaccine**			
1	Yes	186	62.0
2	No	114	38.0
**Willing to definitely accept the HPV vaccine**			
1	Yes	210	70.0
2	No	90	30.0
**Believes they are able to access a clinic/doctor for vaccination**			
1	Yes	240	80.0
2	No	60	20.0
**Believes vaccines are beneficial**			
1	Yes	285	95.0
2	No	15	5.0
**Sources of information**			
1	Mass media/ social media/internet	57	19.0
2	Health workers	117	39.0
3	Workshop/conferences	90	30.0
4	Church	30	10.0
5	Others	6	2.0
**Risk Factors for cervical cancer**			
1	Early sexual intercourse	102	34.0
2	HPV infection	60	20.0
3	Multiple sexual partners	105	35.0
4	Cigarette smoking	18	6.0
5	Kissing	6	2.0
6	I don't knowledge	9	3.0
**Knowledge of HPV prevention measures**			
1	Immunization by HPV vaccine	105	35.0
2	Sexual abstinence	75	25.0
3	Use of condoms	60	20.0
4	Safe sex practices	45	15.0
5	Screening for preinvasive lesions as well as early diagnosis of invasive carcinoma	6	2.0
6	None of the above	9	3.0

Majority of the respondents 80% believes they are able to access a clinic/doctor for vaccination. Majority of the respondents 95% believes vaccines are beneficial. The major sources of information on HPV vaccine were from health personnel (39.0%) and workshop/conferences (30.0%). With regard to risk factors for cervical cancer, the largest risk factors for cervical cancer reported by women were multiple sexual partners (35.5%). In addition, about 34.0% mentioned that early sexual intercourse while 20.0% identified HPV infection. Concerning on knowledge of HPV prevention measures, there was predominantly positive attitude towards the use of HPV vaccination for prevention of cervical cancer among respondents; hence the high rate of desire by mothers to vaccinate their daughters.

Table 3 shows the attitude of mothers toward HPV vaccination of their daughters. Positive attitude prevail in 7 of the 10 attitudinal consideration studied while negative attitude prevail in only 3. Two hundred and seventy two (90.6%) respondents desired to vaccinate their daughters against HPV however only 28 (9.4%) had been able to initiate and/or had completed HPV vaccination for their daughters.

**Table 3:** adolescent mothers attitude towards vaccination of their daughters

Items	Attitude n (%)					
	**SD**	**D**	**U**	**A**	**SA**	**Predominant attitude**
1. HPV vaccine is effective in preventing cervical cancer	15.5%	5.5%	35%	30%	14%	Positive
2. HPV vaccine can cause severe HPV infection	30.5%	15.5%	37%	11.5%	5.5%	Positive
3. Vaccination of adolescent girls will quick earlier sexual activity	30.5%	20 %	35.5%	5.5%	8.5%	Positive
4. HPV may have protracted negative effect on adolescent girls	23%	22.5%	35.5%	10.5%	7.5%	Positive
5. Short-term side effects	9.5%	10.5%	31.5%	30.5%	18%	Positive
6. Availability of HPV vaccine	10.5%	11.5%	30.5%	31.5%	16%	Positive
7. Unknown future side effects	3.0%	12.3%	32.3%	33.7%	18.7%	Positive
8. Cost of HPV is a major barrier to HPV vaccination of adolescent girls	17.0%	15%	30.7%	30.3%	7.0%	Negative
9. HPV vaccination violates cultural norms and my religious beliefs	17.5%	15.0	42.1%	13.2%	12.2%	Negative
10. Safety of vaccine's administration	15.2%	18%	26.7%	30.3%	9.9%	Negative

Out of the 272/300 mothers who had positive attitude to HPV vaccination seen in this study, only 28/300 of them had actually had their daughters vaccinated. Vaccination practice of women with positive attitudinal response to HPV vaccination was significantly lower than those without p=0.02, 95% CI, OR=0.48 (0.90-0.89). Positive attitude significantly led to desire or intention to vaccinate daughters. P-values ranges from 0.00-0.04. The attitude of mothers to the statement “HPV Vaccination may lead to daughters early onset of sexual activity and HPV vaccination violates my cultural and religious beliefs and safety of the vaccine´s administration” was not statistically significant in influencing mothers desire to vaccinate their daughters against HPV. P=0.14 and 0.60 respectively. Only three attitudinal parameters; “vaccination of my daughters will prompt early sexual activity and cost of HPV vaccination being a barrier to vaccination” had statistically significant influence on the practice of HPV vaccination of daughters. Positive attitude to cost of HPV vaccine significantly increased up take of HPV vaccination of daughters while negative attitude to daughters early onset of sexual activity significantly reduced up take. P=0.04 and 0.007 respectively.

Table 4 shows reasons for participating and non-participating of HPV vaccination. About 60.8% and 64.7% of those that had desire to vaccinate, but had not, gave high cost and non-availability of vaccine as their main hindrance. About 90.0% of those respondents that vaccinated their daughters gave fear and life experiences of cervical cancer as the dominant reasons for doing so. Health workers influenced daughters vaccination in 75.5% of cases, while the others (3.0%) were influenced to vaccinate their daughter by their relatives. The average age of daughters at vaccination was 10 ± 0.75 years. Ten (29.6%) of those that initiated vaccination defaulted (Table 4).

**Table 4:** factors influencing practice of HPV vaccination of daughters

Factors influencing practice	Frequency (n=300)	Percent
**A. Reasons for vaccinating my daughter**		
Fear of cervical cancer	272	90.5%
As routine immunization	62	20.75
Life experience of cervical cancer	52	17.3%
To prevent STD	89	29.6%)
Others	9	3.0%
**B. Who influenced the vaccination**		
Health worker	227	75.5%
Relatives	48	15.2%
Friends	28	9.3%
**C. Reasons for not vaccinating my daughter**		
Concerned for side effects	171	56.9%
High cost of HPV vaccine	182	60.8%
Non availability of HPV vaccine	194	64.7%
Fear of promoting sexual promiscuity	272	90.5%

## Discussion

This study describes the knowledge, attitude, desire and practice of HPV vaccination of adolescent girls by their mothers in Eldoret. It provides insights to the mothers in Eldoret's perspectives on whether cervical cancer is preventable and or curable. The introduction of HPV vaccination as a measure to combat cervical cancer in women is well commended but has involved a lot of concern and research. This HPV vaccination essentially targets adolescent girls classed as minors that need parental consent for vaccine administration. The parental awareness, attitude, intention and acceptance of HPV vaccination for their daughters have become relevant for the success of the cervical cancer preventive program. This study found a high level (85.0%) of cervical cancer awareness but much lower awareness of HPV (60.0%) and HPV vaccine (62.0%). Our results were similar to a study done in Kenya by health workers which found out that the level of awareness of cervical cancer to be 91% and 62.7% awareness of HPV vaccine [1, 2, 17].

Being a health worker was one of the main reasons that exposed them to the health information, which explains the higher awareness. While in research conducted by Trim *et al*. 2012 on Parental knowledge, Attitudes, and Behaviours towards HPV Vaccination for their Children, an average of 74.0% of responders knew the relationship between cervical cancer and HPV, only 20.0% of this respondents population identified HPV as a risk factor for cervical cancer [18]. Although the awareness of HPV reported in this study was quite lower than that reported in a study among women health workers in Kenya and the range of 64.7-93.0% reported in a systematic review by Trim *et al*.(2012), the awareness of HPV vaccine in our study was higher than that of the study in review of [1, 2, 17], but was within the range of 47-64.5% reported in the systematic The mean age of the respondents of 34 years found in this study was comparable to 41.3 ± 9.4 years reported in other studies [19, 20].

Majority of the respondents (70.0%) tertiary education, this was in conflict with the results from other studies which found no relationship between level of education and Parental knowledge, Attitudes, and Behaviours towards HPV Vaccination (ibid). However a study in Thailand on maternal acceptance, attitude and knowledge on human papilloma virus vaccination of daughters reported that the basic knowledge of HPV was found to be higher in those that had higher education this results are corresponding to our results which found out that the knowledge of HPV to be higher among the respondents who had achieved post primary education [21]. Two hundred and ten (70%) respondents desired to vaccinate their daughters against HPV however only 90 (30%) that are not willing to accept HPV vaccine.

A study on barriers to HPV immunization for African American adolescent females reported that limited knowledge of HPV connection to cervical cancer led to reduced acceptability [22]. Our study found no statistical significant effects of level of education to acceptability of and practice of HPV vaccination (p=0.969). Despite moderate level of awareness of HPV and HPV vaccine, and poor depth of HPV knowledge, most parents (70%) still indicated intentions to vaccinate their daughters in this study. This was lower than 76% reported in another study and the range of 47-79.5% noted in the systematic review by Trim *et al*. 2012 and Dahlstrom *et al*. 2009 on Attitudes to HPV vaccination among parents of children aged 12-15 years a population-based survey in Sweden [18, 23]. On the whole, this study found a typically positive attitude towards HPV vaccine among mothers of adolescent girls in Eldoret, Kenya. This led significantly to high intent to vaccinate their daughters. Only in three parameters out of ten parameters did negative attitude dominate their intention.

This finding, thus consistent with a study done by Pinto and colleagues, which also found out that the intent to vaccinate was directly related on attitude and apparent behavioral control. Positive attitudes nevertheless do not at all times lead to lead to parents accepting their daughters to be vaccinated [24]. This possibly reflected the influence of other factors on mothers participating in HPV vaccination for their daughters. Positive attitude to cost significantly influenced participating in HPV vaccination where vaccination is free to adolescent girls aged 10. This is quite applicable in developing countries with low resource such as Kenya where many mothers may not have the funds for the HPV vaccine. Only 28 (9.4%) of the respondents had initiated vaccination for their daughters in this study. Though this is lower but it is comparable to the 19.0-21.0% participants rate reported in other studies [19, 25, 26].

The fear for cervical cancer, and life experiences of cervical cancer are the predominant reasons for mothers of adolescent girls to participate in HPV vaccination. Participating in HPV vaccination in our population while lack of awareness of HPV vaccine, concern about efficacy and adverse side effects of HPV vaccine, high cost of HPV vaccine and young age of their adolescent girls were explanation given by mothers that did not vaccinate their daughters. This concurred with other studies that identified fear of side effects of vaccine, perceiving the HPV vaccine as risky and belief that vaccine is experimental, low knowledge of HPV vaccine and cervical cancer and not participating in routine cervical smear as reasons for reduced intention to vaccinate and participating in HPV vaccination for their adolescent girls [19, 21, 27].

## Conclusion

Even though the results of this study may have limited generalized, they provide an insight into the knowledge, attitude, desire and practice of HPV vaccination of adolescent girls and factor that determine intent and practice of HPV vaccination of adolescent girls by their mothers in Eldoret from the parental perspective. We conclude that the awareness of HPV and HPV vaccine for prevention of cervical cancer is still suboptimal among mothers of adolescent daughters in our environment. There was predominantly positive attitude towards the use of HPV vaccination for prevention of cervical cancer among respondents; hence the high rate of desire by mothers to vaccinate their daughters. The practice of the HPV vaccination by mothers of adolescent girls was still low. There is need for increased awareness creation by government agencies and caring physicians. The government departments responsible for routine immunization should latch on this positive attitude by mothers to increase uptake by making HPV vaccination part of routine immunization for the adolescent. There is need for policy to guide and unify various stake holder efforts in the struggle against an increasing cervical cancer burden. On the other hand the policy will also save as a legal framework for service users, NGOs and activists to base their arguments on, when advocating for more financial and technical support from government and its partners that are playing different roles in combating cervical cancer in Kenya.

### What is known about this topic

- Cervical cancer though preventable is a leading cause of mortality worldwide;
- Human papillomavirus is the main cause of cervical cancer and is preventable through vaccination.

### What this study adds

- This study provides an insight into the knowledge, attitude, desire and practice of HPV vaccination of adolescent girls;
- The study also shows the factors that determine intent and practice of HPV vaccination of adolescent girls by their mothers from the parental perspective.
